# A Quantitative Assessment of Trainers-Dolphins’ Interactions in the Absence of Food Reward

**DOI:** 10.3390/ani13101651

**Published:** 2023-05-16

**Authors:** Sara Platto, Agathe Serres

**Affiliations:** 1 Department of Biotechnology, College of Life Sciences, Jianghan University, No. 8, Sanjiaohu Road, Wuhan Economic & Technological Development Zone, Wuhan 430056, China; 2 Institute of Deep Sea Science and Engineering, Chinese Academy of Sciences, 28 Luhuitou Road, Jiyang District, Sanya 572000, China

**Keywords:** human-animal interaction, human-animal-relationship, dolphins’ object play, trainer-dolphin interaction, bottlenose dolphins’ welfare

## Abstract

**Simple Summary:**

In general, it is difficult to assess the motivation of dolphins when participating in training sessions since most of these animals’ activities with their trainers are reinforced with food rewards. The current study was carried out at The Dolphin Reef (Eilat, Israel), a unique place where the interactions between the trainers and the dolphins were dissociated from the food reward. The dolphins showed high participation rates and promptness (short latency of response) in responding to the trainers’ presence on the platforms or in the water with or without the “call” (dolphins often anticipated the beginning of the sessions or arrived at the trainers’ location in less than a minute). Diel and seasonal differences were also recorded, with more dolphins participating during the morning sessions and the neutral seasons (i.e., time of the year without mating activities and births). Differences in the participation rates, depending on the trainers, might also indicate a preference of the dolphins toward a specific caretaker. In addition, inter-individual differences in the dolphins’ participation in the trainers’ sessions were also recorded, with potential links with the animals’ personality and/or health status depending on the specific case. The results discussed in this paper suggest that the trainer dolphin interactions (TDIs) may be an important part of the captive dolphins’ life, and for this reason, they should be closely monitored in order to improve the social environment of these animals under human care and thus their welfare.

**Abstract:**

All the studies that have considered the motivation of the dolphins to interact with their trainers as a possible welfare indicator have been carried out in facilities where the trainer-dolphin interactions (TDIs) sessions were reinforced with food. Therefore, in these specific circumstances, it was difficult to separate the motivation of the dolphins interacting with the trainers from the food drive. The current study aims to assess the interaction between the trainers and the dolphins in the absence of food rewards. The research was carried out at The Dolphin Reef (Eilat, Israel), a facility where the interaction between the trainers and 14 bottlenose dolphins of different sex and age classes did not involve food rewards. A total of 531 TDIs were recorded, with dolphins participating in 94.5% of the sessions and an average of three dolphins per session. The dolphins participated in a higher number and more frequently in the TDIs when toys were provided by the trainers. Diel and seasonal differences were also observed, with the dolphins participating more during the morning sessions and the neutral season. The latency of response of the dolphins to the presence of the trainers on the platform or in the water, whether or not advertised by the trainers’ signal (“call” or “no-call”), was very short (usually less than 1 min), and the dolphins often anticipated the beginning of the sessions by arriving at the trainers’ location before or upon the caretakers’ arrival (96% of the time). Individual differences in the participation in the TDIs were also recorded, which might be linked to both the dolphin’s health/welfare status or their personality. The current study shows that the separation of the TDIs from the food reward allows for a better understanding of the willingness of dolphins under human care to interact with their trainers. In addition, the results presented in this paper show that such TDIs are an important part of these dolphins’ lives, which suggests that these interactions might be an additional tool to improve the animals’ social environment and monitor their welfare.

## 1. Introduction

Humans and non-human animals encounter each other in a variety of settings that create opportunities for interactions to take place. The study of human-animal interaction (HAI) has been conducted on different animal species, such as companions, farms [1,2], laboratories [3], zoos [4], and even wild animals [5]. An interaction between two parties can be defined as “*a sequence in which individual A shows behaviour X to individual B, or A shows X to B, and B responds with Y*” [6,7]. When repeated interactions between the same animals and humans occur, these can lead to the development of a human-animal-relationship (HAR) [6,8,9], or in the case of positive interactions, of a human-animal-bond (HAB), which can influence the behavior and well-being of the involved parties [10]. In facilities such as zoos, farms, and aquariums, HAIs happen routinely, and they can have a positive or negative impact on the animals’ well-being depending on the nature, duration, and regularity of the interactions, but also on the personality and the emotional/affective states of the animals and caretakers involved [11,12,13,14]. For these reasons, the study of HAIs has been shown to be a critical and useful element in assessing the welfare of different animal species under human care [2,10,15,16,17,18,19,20,21,22]. Many studies have been performed on HAIs in domesticated animal species [23], but research on captive wild animals is still limited, despite some reports on zoo animals [4,7,24,25,26,27,28,29], including dolphins [30,31,32,33,34].

The development of a HAR requires the animals to recognize the human companion, comprehend his/her gestures, and understand his/her attentional and emotional states [14]. Dolphins have already been shown to possess these cognitive characteristics, as they recognize their trainers individually using visual cues, understand their attentional states, and can follow several types of human pointing gestures [14,35,36,37,38]. Among dolphin species under human care, bottlenose dolphins (*Tursiops truncatus*) are usually subjected to daily training that involves close contact with familiar trainers, which often can lead to the development of HARs [14]. The effects of these activities on the dolphins’ behavior have been the focus of several welfare studies, with some concluding the animals view the training sessions positively [39,40], while others suggest a negative impact on the animals’ well-being [30]. Recently, the behavior of animals during training sessions, especially their motivation to interact with their caretakers, has been put forward as a potential welfare indicator [41,42]. However, since most of the activities conducted with captive dolphins, including training, are reinforced by food rewards that are part of the animals’ daily meal rations [43,44], it is difficult to assess the motivation of the dolphins to interact with the trainers and separate it from the animals’ food drive.

For animals such as bottlenose dolphins that live in a fission-fusion social group system, where individuals disperse and regroup on a daily or hourly basis [45,46], the maintenance of social bonds occurs through affiliative behaviors such as synchronous swimming, body contact, and play activities [47,48,49]. Dolphins’ skin is very sensitive, with a high concentration of nerve endings in different parts of the body [50], explaining why they use body contact in social contexts. It has been suggested that physical contact with humans may be as motivating as social interactions with conspecifics for some animal species [51]. In captivity, dolphins have been observed making persistent attempts to be rubbed and petted, so lately, this behavior has been used as a secondary reward by the trainers [52,53]. Similarly, play behavior is also present among different cetacean species, including bottlenose dolphins, and it is used by young as well as mature individuals in social contexts to solidify bonds and gain knowledge about conspecifics’ status [49,54]. Wild dolphins have often been observed manipulating objects encountered in their environment, such as logs, kelp, feathers, and even animal species such as jellyfish and fish [55], while under human care, dolphins play with man-made-objects provided by the trainers [55,56]. Inter-species play behavior between dolphins and humans has also been witnessed in the wild, where dolphins engaged in loose play activities with humans that consisted of the exchange of natural objects [57]. In captive settings, the presence of the trainers seems to be more enriching for bottlenose dolphins than objects [58], resulting in an increase in play behaviors among the dolphins involved in the activity [39]. Inter-specific body contacts (i.e., petting) and object play often occur during interactions between trainers and dolphins, which could play a role in the development of the dolphin-trainer relationship. Therefore, investigating the motivation of dolphins to engage in these activities may provide additional information about the development of the HAR.

As mentioned earlier, activities between trainers and dolphins are usually reinforced with food rewards, which makes it difficult to understand if the animals are really interested in interacting with the trainers or if the food drive represents the primary motivation. The current study was carried out at The Dolphin Reef (TDR) in Eilat (Israel), a unique dolphin facility where training/interaction sessions and feeding are separated, and where food rewards were never provided during the interactions with the trainers, resulting in voluntary participation by the dolphins. Trainers would only use secondary reinforcements such as vocal cheers, hand clapping, and petting to reward dolphins during training sessions [59,60,61]. The current study aims to quantitatively assess the trainer-dolphin interactions (TDIs) without the influence of food rewards. The following two possible hypotheses are drawn: (1) the bottlenose dolphins are willing to interact with familiar humans (i.e., trainers) even in the absence of food rewards; (2) the participation of the dolphins in the sessions depends on the animals’ characteristics, context, and the trainer’s identity. The potential link between dolphins’ health and welfare, and their participation in the TDIs will be further discussed using individual cases. This study represents the first insight into the non-food-driven HAI in bottlenose dolphins under human care.

## 2. Materials and Methods

### 2.1. Settings

**Figure 1:**
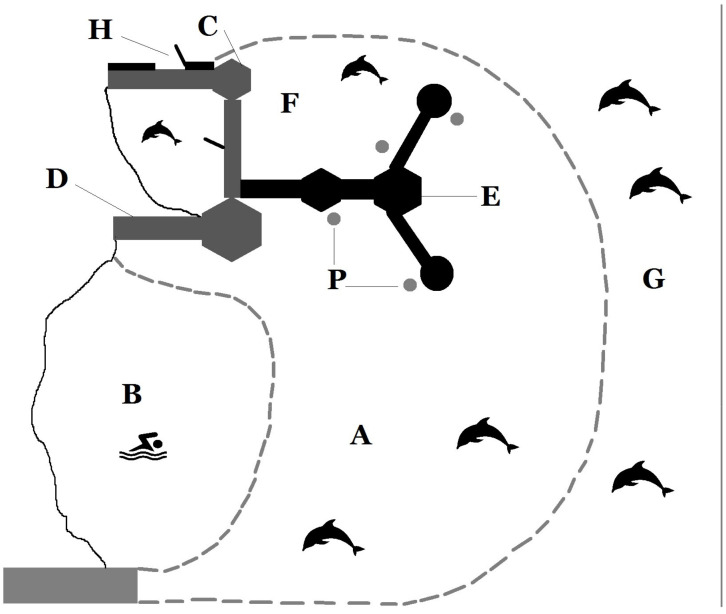
Map of The Dolphin Reef site: (A) dolphins’ enclosure; (B) tourists area; (C) trainers office; (D) observation tower; (E) I epsilon pier where tourists could walk and sit to interact with the dolphins; (F) small enclosure where the two males and two females were separated from the rest of the group from 9 am to 4 pm every day; (G) open sea; (H) location of the gate that gave the dolphins access to the open sea; (P) platforms where the trainers performed the sessions with the dolphins.

The study was conducted at "The Dolphin Reef” (TDR) (Eilat, Israel), located in the northern region of the Gulf of Aqaba (Red Sea). The facility was a semi-confined marine enclosure surrounded by nets, with a surface area of about 100 ha (14,000 square meters), which hosted 14 individuals of bottlenose dolphins of different ages and sex classes. The depth of the water inside the enclosure varied from 0 m at the shore to 12 m at the perimeter net. When the facility was set up, a 24-h open gate to the sea was located on one side of the enclosure, which enabled the dolphins to access the open sea and return to the enclosure without any restrictions. The gate was later closed following conflicts between TDRs dolphins and the local aquaculture facilities (trainers’ personal communication). By the time the current study began, the dolphins had already become habituated to not having access to the open sea. The site was characterized by relatively clear water and favorable weather conditions for most of the year, which allowed us to identify individual dolphins using well-recorded visual characteristics (dorsal fin shape, scratches, marking, behavioral patterns). A research laboratory tower located on the perimeter of the enclosure about 10 m above sea level provided an ideal vantage point to observe the dolphins. Four small round platforms located around the enclosure were used by the trainers to interact with the dolphins (Figure 1).

### 2.2. TDR Management

TDR was primarily a recreational tourist site, and its management policy differed from the one usually adopted in other traditional dolphinariums. In fact, there was no direct link between the dolphin feedings and the training/interactions, which had been a policy since the facility was established. As a result, the dolphins voluntarily cooperated with their trainers even outside of feeding sessions. The training sessions were performed within the TDIs (during the intervals between each feeding time), and the trainers used stroking, hand clapping, or voice cheering as rewards for successful performances instead of fish. The training sessions were performed by using positive reinforcement techniques, where the trainers shaped or captured new behaviors of the dolphins, or reinforced previously learned tasks. TDR was not a facility where dolphins were trained to perform in shows. However, during TDIs, trainers could also educate the public on dolphins’ biology and behavior, swim in the facility to assess the social situation of the animals, or provide human-made objects (toys) for the dolphins to play with. As the current study did not aim to assess the success rate of the dolphins’ training without food rewards, no data on the matter are reported here. 

Five feeding sessions usually took place every day. The first feeding time was performed at 9 am (with a duration of 15 min or less) while the facility was closed to the public, and it was used to monitor the health of the dolphins and for medical training. This session was the only occasion where training was reinforced with food. Other feeding sessions occurred at 10 am, 12 pm, 2 pm, and 4 pm, with the dolphins divided into four groups, each fed at a different platform. After the last feeding time of the day, the dolphin area was closed to the public, and no interaction with the trainers were performed. The feeding sessions were only meant to provide food to the dolphins; therefore, no training or any other type of interactions with the trainers occurred during this time. The activities involving the presence of the public started at 10 am, after the second food provisioning, and ended at 4 pm after the last meal, and they included the following:

1. *Snorkeling and diving*: tourists could perform a swim inside the dolphins’ enclosure accompanied by an instructor;
2. *Dolphin-therapy sessions*: disabled people (children and teenagers) accompanied by a trainer underwent an interaction with the dolphins;
3. *Tourists-dolphins’ interactions*: tourists could walk on the epsilon-shaped platform and sit to look for interactions with the dolphins.

All TDIs sessions occurred during the intervals between each food provisioning: 10.15–11.45 a.m.; 12.15–1.45 p.m.; 2.15–3.45 p.m.

### 2.3. Study Subjects

The dolphin group living at TDR was established in the 1990s with the following two adult males (Cindy and Diky) and three females: one adult (Shy) and two juveniles (Domino and Dana), which were brought from the Black Sea (Russia). One male from the founding group, Diky, was returned to the Black Sea in 1996, while the rest of the subjects were born at TDR. The individuals belong to the subspecies Black Sea Bottlenose Dolphin (*Tursiops truncatus ponticus*), endemic to the Black Sea, and isolated from other populations of bottlenose dolphins in the Mediterranean Sea and other waters [62]. These subspecies present the same external characteristics as the other close bottlenose dolphin subspecies, *Tursiops truncatus aduncus*, which is common in the Red Sea. TDRs dolphins were still able to eat fish they hunted, and they were sometimes observed refusing the fish provided by the trainers after catching the fish that escaped from the neighboring fish farms.

**Figure 2:**
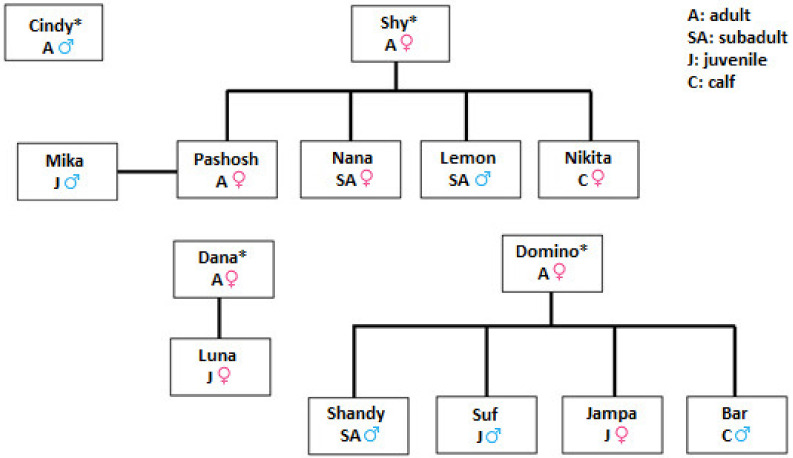
Features and maternal lineages of the dolphins housed at The Dolphin Reef. Individuals denoted with * were transferred in 1990 from the Black Sea and composed the initial group. All other individuals were born at TDR.

TDRs dolphin group included individuals of different sex and age classes (Figure 2). At the beginning of the study, the group of dolphins was composed of 12 individuals that reached the number of 14 with the birth of Bar and Nikita in the summer. Another female calf was born from Pashosh in the same summer but died immediately after the delivery. During the study period, the group experienced the following changes:

1. Mika (female juvenile), Domino (adult female), and Bar (Domino’s calf) died, seven, eight, and nine months after the beginning of the study, respectively;
2. At the start of the study, all dolphins were housed together in the same enclosure, but two months later, because of the continuous fights between the dominant male (Cindy) and the two adult males (Shandy and Lemon), a new sub-enclosure was built inside the existing one. During the day, from 9 a.m. to 4 p.m., the two adult males (Shandy and Lemon) were confined in this new area. After the last food provisioning (4 p.m.), they were free to join the rest of the group. Two adult females (Nana and Pashosh) were added to this new enclosure five months later Lemon and Shandy, to create a stable sub-group with the aim to release them back to the Black Sea.

The age class was arranged into the following two groups: Adults and Young (including juveniles and calves).

### 2.4. Trainers and TDIs

The following four trainers participated in the study: Delicia, Hen, Tal (women), and Ouri (man). These trainers were familiar with and to the animals since they had been working at TDR for at least four years, and they also were the same people who fed the dolphins. The trainers followed a specific timetable and spent a total of three hours daily in the TDIs on the platforms or in the water differently distributed throughout the day. This also included a long swim around the facility to check the status of the dolphins’ group and their activities. The TDI represented the only moment the trainers interacted with the dolphins outside of the feeding times. The trainers could choose the location, time, duration, and type of activity to propose to the dolphins within the TDIs. Each trainer had an object (e.g., two metal sticks or several linked shells) that they shook in the water to produce a sound wave that represented the trainer’s identity. The association between the sound produced by the object and the trainer’s identity was adopted since the setup of the dolphin’s facility, and it was conditioned when each trainer started to work at TDR. The trainers could use their object to “*call*” the dolphins (i.e., advertising the trainers’ presence and availability to interact with the animals) by shaking it in the water at the beginning of the TDIs sessions. Sometimes, the dolphins were already waiting for the trainers at the platform or arrived at the location immediately upon the caretakers’ arrival, and in this situation, the “call” was not necessary (the situation hereafter referred to as “*no-call*”). This also often occurred when the trainers were engaged in conversations with the public upon their arrival at the platform (delivering information regarding the dolphins’ biology and behaviors to the public), which might have given the dolphins the time to approach the trainer’s location before the “call” was performed. Since no food was provided during the TDIs sessions, the dolphins were “free” to choose whether or not to interact with the trainers and for how long.

In general, the modality on how TDIs were performed mostly followed the personality of each trainer. Precisely, more “dynamic” interactions were characterized by training sessions or playing activities with the trainers jumping in the water or from one platform to another one, while in more “nurturing” sessions, the trainers were focused on delivering petting upon “dolphins request”. In fact, it could often happen that the dolphins approached the trainers at the platform by immediately showing their belly-up, which was interpreted by the caretakers as a desire of the animals to be petted.

Only two trainers (Chen and Delicia) offered to perform play sessions with toys during the TDIs. During the play sessions with toys, the trainer would place the toy in the water to present it to the dolphin(s) and wait for the animals to interact with it. A *play activity* was considered to happen when a dolphin engaged in an interaction with the toy alone (by tossing around the object with the head, tail, or pectoral fins), with other dolphins, or with the trainers. The object play among the dolphins mostly involved chasing each other to obtain the toys, while the play activities between trainers and dolphins were characterized by play-fetch (the dolphins brought the toy back to their trainer), tag-of-war, or chasing after the trainer to take the object. The modality of the play activities was left to the experience of the trainers to engage the dolphins with the toys. The data collected during the object play sessions are further discussed in the “*Data collection*” session. 

### 2.5. Data Collection

The data collection was carried out over a period of 13 months, during which the following three different seasons were considered: the breeding season (February–May), the delivering season (June–August), and the neutral season (September–January), the latter indicating the time of the year where no births or breeding activities occurred. Only the trainer-dolphin-interaction (TDIs) sessions were monitored during the following intervals between each food provisioning from after 10 a.m. to before 4 p.m.: 10.15–11.45 a.m.; 12.15–1.45 p.m.; 2.15–3.45 p.m. A total of 531 sessions were observed over 162 days. A session started when a trainer arrived on the platform and either performed the “call” to inform the dolphins of his/her availability to interact with them or not if the animals had already approached the platform upon the caretaker’s arrival. The interaction area was defined as the space within 5 m from either the platform where the trainers stood, or from the trainers in the water (approximately two dolphin’s body lengths [63]). In addition, the total number of dolphins participating in the sessions as well as each time a “call” or “no-call” (the trainers did not use the “call” to advertise their presence) was followed by the arrival of a dolphin, and the “latency response” (i.e., the interval between the start of a “call” or “no-call” and the first dolphin/s to enter the interaction area) were recorded. The latency response was categorized using six time intervals ranging from “0” (the dolphin approached the platform immediately upon the trainer’s arrival) to “over 10 min” (Table 1). 

Further monitoring of the TDIs was performed using the following three different recording methods:

1. *Scan sampling*. A total of 289 TDIs sessions without toy(s) were monitored using scan sampling over a period of 10 months. The scan interval time was 20 s so that whenever several dolphins approached the trainer at the same time, there was enough time to record each individual’s behavior. The behavior of the dolphins was scored following a specific ethogram (Table 2).
2. *Continuous recording and event sampling*. A total of 150 TDIs sessions without toy(s) were monitored using continuous recording, while 92 TDIs sessions with toy(s) were monitored using event sampling for a total of 242 sessions over a period of 13 months. The sessions with toys were video-recorded using a video-camera VP-L750 Hi 8 PAL with video-8 tapes. The behaviors of the dolphins were scored following a specific ethogram (Table 3 and Table 4).

**Table 1:** List of latency criteria for the response of the first dolphin to the beginning of a TDI session.

Latency	Description
0	The dolphin is already present next to the platform when the trainer starts the session, or the dolphin approaches the trainer when he/she is still performing the “call”. The same interval was also considered when “no-call” was performed.
0–1	The dolphin approaches the trainer within an interval from 0 to 59 s after the “call” was performed. The same interval was also considered when “no-call” was performed.
1–2	The dolphin approaches the trainer within an interval from 1 min to 1 min and 59 s after the “call” was performed. The same interval was also considered when “no-call” was performed.
2–5	The dolphin approaches the trainer within an interval from 2 min to 5 min and 59 s after the “call” was performed. The same interval was also considered when “no-call” was performed.
5–10	The dolphin approaches the trainer within an interval from 5 min to 10 min after the “call” was performed. The same interval was also considered when “no-call” was performed.
Over 10	The dolphin approaches the trainer more than 10 min after the “call” was performed. The same interval was also considered when “no-call” was performed.

**Table 2:** Ethogram of the behaviors observed during the TDIs sessions without toys provided by the trainers and collected using scan sampling method.

Behavioral Category	Behavior	Description
Training	Training	All activities guided by the trainers that involved teaching/requesting the dolphins a new behavior, with petting or hand clapping used as reinforcement.
Playing	Playing	A play activity initiated by the dolphins bringing to the trainer objects (a piece of plastic, a leaf, or dead jellyfish) they found in the enclosure.
Waiting	Waiting	A dolphin approaches the trainer who is already engaged in an activity with another dolphin, and waits for the trainer’s attention.
Petting	Petting	The trainer strokes a part of the body of the dolphin.
Other Activities	Approach	Dolphin approaches the trainer who is on the platform or in the water.
	Leaving	Dolphin leaves the interaction area.
	Swimming Around	Dolphin swims around within the interaction area but without approaching the trainer.
	Passing-by-and-looking	Dolphin passes through the interaction area without approaching the trainer, but making eye contact with the caretaker.
	Staying under the platform	Dolphin stations under the platform where the trainer is located.
	Jumping around	Dolphin jumps around within the interaction area without approaching the trainer.

**Table 3:** Ethogram of the behaviors observed during the TDIs sessions with and without the toys provided by the trainers, and collected using continuous recording sampling method.

Behavioral Categories	Description
Object Play	The trainer initiates play activities with toy(s)
Other Activities	All other activities (swimming around; passing-by-and-looking; staying under the platform; jumping around; waiting; playing; petting; training)

**Table 4:** Ethogram of the behaviors observed during TDIs sessions with toy(s) provided by the trainers and recorded using event sampling method.

Behavioral Categories	Type of Behavior	Description
Object Play	Social Play-Trainer	Play activity involving toy(s) between the trainer and a dolphin. During the play activity, the dolphin surrenders the toy to the trainer.
	Social Play-Dolphin	Play activity involving toy(s) among two or more dolphins. During the play activity, one dolphin surrenders the toy in favor of another individual.
	Individual Play	A dolphin interacts alone with the provided toy(s).
	Stealing Toy	One dolphin takes the toy, used by another dolphin, away from the interaction area
	Other	Any other behavior not included in the above categories (swimming around; passing-by-and-looking; staying under the platform; jumping around; waiting; playing; petting; training)
Interaction initiation and ending	Initiation of Interaction	Indicates when either the trainer or one dolphin initiates the play activity
	End Interaction	Indicates when either the trainer or one dolphin ends the play activity by leaving the interaction area, or by starting another activity.

### 2.6. Statistical Analysis

For the statistical analysis, the dolphins were categorized into the following two age classes: adults and young (including juveniles and calves). First, the participation rates for each dolphin with the trainers during the TDIs were calculated by dividing the number of sessions each dolphin participated to by the total number of sessions the dolphin could participate in (i.e., the dolphin was alive and had access to the platform where the trainer stood). The rates of the time spent by each dolphin with the trainers were also calculated by dividing the total time spent by each dolphin with the trainers during the data collection by the total duration of the sessions the dolphin could participate in. 

All the statistical analysis was conducted in R 4.0.5 (R Core Team, 2020). Because of the different methods used during the data collection, the analyzed data are represented by duration in seconds (continuous recording), the proportion of time (numbers of scans), and event sampling (frequency). The statistical analysis was conducted according to the type of data.

#### 2.6.1. Dolphins’ Overall Participation to the TDIs Sessions

The effect of the context of the session on the participation of the dolphins was analyzed using a generalized linear mixed effect model (GLMM) for binomial data (glmer() function from the lme4 package [64]). The response (participation/no participation) was used as the response variable, and the type of session (with or without toys), time, season, trainer ID, call modality (“call” and “no-call”), and call duration as predictors. The session ID and dolphin ID were included as random factors.

The effect of the context of the session on the number of dolphins participating in the session was analyzed using a linear regression (lm() function from the “stats” package) with the number of dolphins participating as the response variable and the type of session, time, season and trainer ID as predictors.

#### 2.6.2. Latency of Response

The latency of response of the dolphins to the trainer’s “call”, or to the caretakers’ presence on the platform or in the water (“no-call”) was transformed into an ordinal variable (0: 0 min, 1: 0–1 min, 2: 1–2 min, 3: 2–5 min, 4: 5–10 min, 5: >10 min), and the absence of response was coded as not applicable (NA). The effect of the context of the session and the dolphins’ features on the latency of response was analyzed using one-way repeated ordinal regressions (clmm() function from the ordinal package [65]). The latency of response was used as the response variable, and the type of session, time, season, trainer ID, call modality, call duration, dolphin age, and sex as predictors. The session ID and dolphin ID were included as random factors (Table 5).

#### 2.6.3. TDIs Sessions without Toy(s) (Scan Sampling)

The effect of the context of the session and dolphins’ features on the number of training scans, contact scans, petting scans, playing scans, waiting scans, and “other” activities scans were analyzed using GLMMs for Poisson distributed data with the number of scans spent in each activity as the response variable, and the time, season, and trainer ID, dolphin age and sex as predictors. The session ID and dolphin ID were included as random factors, and the total number of scans was included as an offset. One model was run for each activity.

#### 2.6.4. TDIs Sessions with Toy(s) (Continuous Recording)

The effect of the context of the session and dolphins’ features on the duration of the play activities during the TDIs sessions with toy(s) was analyzed using linear mixed effect models (LMMs, lmer() function from the lme4 package [64]) with the time spent playing by the dolphins as the response variable, and the time, season, trainer ID, type of object play (social play-dolphin-trainer, social play-dolphin, individual play, stealing object), initiation and ending modality (dolphin or trainer), dolphin age and sex as predictors. The session ID and dolphin ID were included as random factors, and the session duration was included as an offset.

A diagnosis was performed on each model, including normal distribution, homogeneity of variances, the goodness of fit and multi-collinearities for linear regressions, normal distribution and homogeneity of variances, and multi-collinearities for LMMs, homogeneity of variances, and multi-collinearities for GLMMs. Multi-collinearities were checked using a variance inflection factor (VIF) with no major issues (no VIF > 3). Wald chi-squared tests were used to obtain *p*-values from all models. Pairwise tests were conducted by running the same models with appropriate sub-settings and applying a Bonferroni correction. Fitted data from models were plotted using the sjPlot package [66].

## 3. Results

The dolphins participated to 94.5% of the sessions (502 over 531), with only 5.5% of sessions (29 sessions over 531) with no participation at all. The bottlenose dolphins participated slightly more in sessions with toy(s) (96%) than in sessions without toy(s) (94.3%). Participation was the highest during the neutral season (56.3%), followed by the breeding (22.6%), and delivering season (21.1%). Participation was also the highest at noon (35.6%), followed by the afternoon (34.3%), and the morning (30.1%). 

The trainer with the highest number of monitored sessions was Chen (180: 33.9%), followed by Delicia (171: 32.2%), Tal (109: 21%), and Ouri (71: 13.4%). The trainer with the highest percentage of sessions involving “calling” was Delicia (81.1%), followed by Chen (76.2%), Tal (58.5%), and Ouri (55.8%).

### 3.1. Individual Differences in the Participation to the TDIs

Individual differences in the participation in the TDIs sessions were reported. Mika and Shy exhibited the lowest rates of participation, and total time spent with the trainers (Table 6). On the other hand, Shandy, Lemon, and Suf exhibited the highest rates of participation, and/or total time spent with the trainers.

### 3.2. Overall Dolphins’ Participation in the TDIs

The number of dolphins participating in the sessions varied from 0 (5.5% of sessions) to 10 (0.7% of sessions), with an average of 3 dolphins per session (3.54 ± 0.094). Females participated more than males (84.7% versus 81.4% of the sessions), and adults participated more than young individuals (85.5% versus 61% of the sessions). 

**Figure 3:**
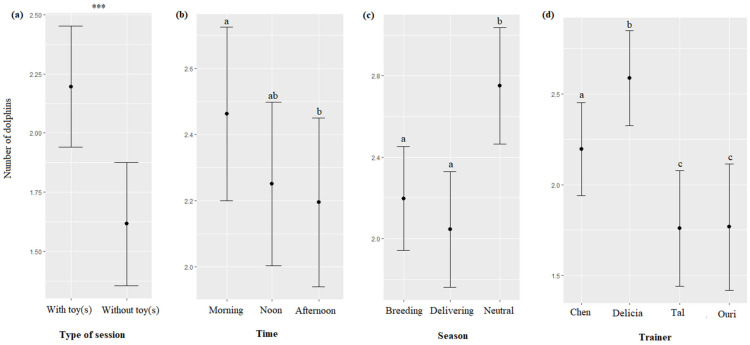
Means and 95% confidence intervals of the number of dolphins participating in sessions depending on (**a**) the type of session, (**b**) the time, (**c**) the season, and (**d**) the trainer’s ID. ***: *p* < 0.0001, for variables with more than two levels, different letters indicate significant differences (Wald chi-squared test with Bonferroni correction).

Significantly more dolphins participated in the sessions involving toy(s) than in sessions not involving toy(s) (F = 23.18, df = 1, *p* < 0.0001, Figure 3a). The time of the session impacted their participation (F = 3.20, df = 2, *p* = 0.0410), with more dolphins participating in the morning than in the afternoon (Table 7, Figure 3b). The season impacted their participation (F = 28.07, df = 2, *p* < 0.0001), with more dolphins participating in the neutral season than in the delivering and breeding seasons (Table 7, Figure 3c). The identity of the trainer impacted their participation (F = 22.75, df = 3, *p* < 0.0001, Figure 3d), with more dolphins participating with Delicia, followed by Chen, and Ouri and Tal (Table 7).

### 3.3. Frequency of the Dolphins’ Response to the “Call” and “No-Call” of the Trainers

The trainers performed the “call” during 76% of sessions to advertise their availability to interact with the dolphins, of which 66% of the times resulted in the dolphins approaching the trainers. On the other hand, when trainers did not perform the call, the dolphins approached the platform 96% of the time. 

**Figure 4:**
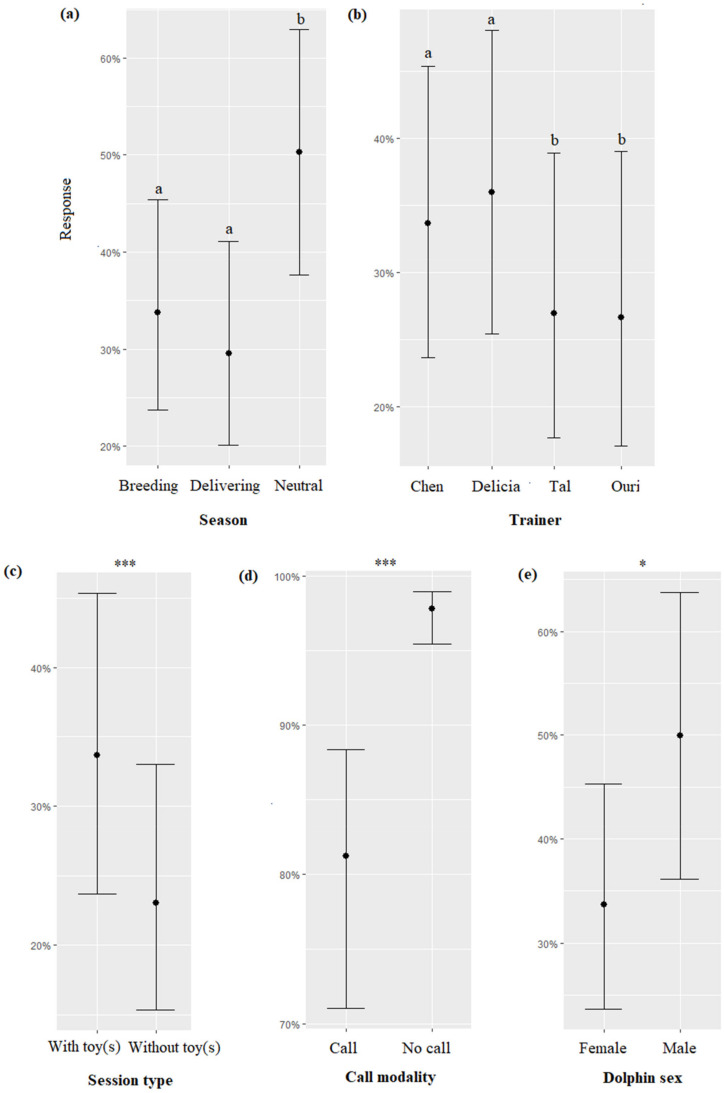
Means and 95% confidence intervals of the response of dolphins to sessions depending on (**a**) the season, (**b**) the trainer, (**c**) the type of session, (**d**) the call modality, and (**e**) the dolphin sex. *: *p* < 0.05; ***: *p* < 0.0001; for variables with more than two levels, different letters indicate significant differences (Wald chi-squared test with Bonferroni correction).

The season, the session type, the trainer’s identity, the call modality, and the sex of the dolphins significantly impacted the response of dolphins (respectively, χ^2^ = 64.15, df = 2, *p* < 0.0001; χ^2^ = 16.28, df = 1, *p* < 0.0001; χ^2^ = 14.68, df = 3, *p* = 0.002; χ^2^ = 5.58, df = 1, *p* = 0.018). Overall, dolphins responded significantly more to the presence of the trainers on the platform independently from the modality of the “call” during the neutral season than during the delivering season and the breeding season (Figure 4a, Table 7), more when the trainer was Delicia or Chen than when it was Tal or Ouri (Figure 4b, Table 7), more when toy(s) were provided than when not (Figure 4c), and male dolphins responded more often than females (Figure 4e). The dolphins approached the trainers more frequently when “no call” was made than when a “call” was performed (Figure 3d). The time, duration of the “call”, and dolphin age did not significantly influence the response of dolphins (respectively, χ^2^ = 3.73, df = 2, *p* = 0.1546; χ^2^ = 0.45, df = 1, *p* = 0.7976; χ^2^ = 2.24, df = 1, *p* = 0.1344).

### 3.4. Latency of Response of the Dolphins to the “Call” and “No-Call” of the Trainers

When the trainers performed the “call”, dolphins responded more frequently with an interval time of 0–1 (from 1 s to 59 s: 22.6%). Similarly, when the trainers did not perform the “call”, dolphins responded more frequently with an interval between 0 (25%) (indicating an immediate arrival of the dolphins upon the arrival of the trainers on the platform) and 0–1 (26%). When assessing the first dolphin responding to the trainer’s presence on the platform/in the water independently on the modality of the “call”/”no-call”, the sex-age class that responded first was represented by adult females (22.1%), followed by adult males, and young females (both 19.6%), and young males (12%).

**Figure 5:**
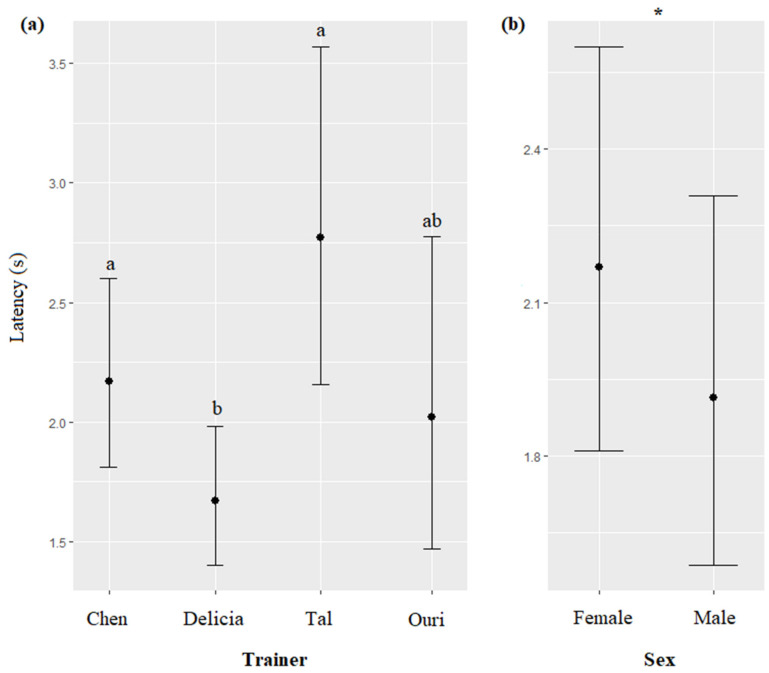
Means and 95% confidence intervals of the latency of the first dolphin to arrive at the platform depending on (**a**) the trainer, and (**b**) the dolphin’s sex. *: *p* < 0.05, for variables with more than two levels, different letters indicate significant differences (Wald chi-squared test with Bonferroni correction).

The trainer’s identity and the sex of the dolphin significantly impacted the latency of response of the dolphins (respectively, χ^2^ = 27.96, df = 3, *p* < 0.0001; χ^2^ = 4.09, df = 1, *p* = 0.0430). Dolphins responded significantly quicker when the trainer was Delicia than when it was Tal or Chen (Figure 5a), and females responded slower than males (Figure 5b, Table 7). The type of session, time, season, call modality, duration of the call, and age of the dolphin did not significantly influence the latency of response of dolphins (respectively, χ^2^ = 0.66, df = 1, *p* = 0.4153; χ^2^ = 1.88, df = 2, *p* = 0.3902; χ^2^ = 4.12, df = 2, *p* = 0.1268; χ^2^ = 0.70, df = 1, *p* = 0.4021; χ^2^ = 0.59, df = 1, *p* = 0.4401; χ^2^ = 0.01, df = 1, *p* = 0.9347).

### 3.5. TDIs during Sessions without the Toy(s) Provided by the Trainers

During TDIs sessions where trainers did not provide toy(s), dolphins spent most of the time engaged in “other activities” (34.9% of scans), followed by “petting” (27.8%), “waiting” (18.8%), “training” (16.7%), and “playing” (1.8%). During these sessions, play activity was initiated by the dolphins who brought the trainers pieces of plastic or dead jellyfish they found in the facility. 

#### 3.5.1. Training Activity

**Figure 6:**
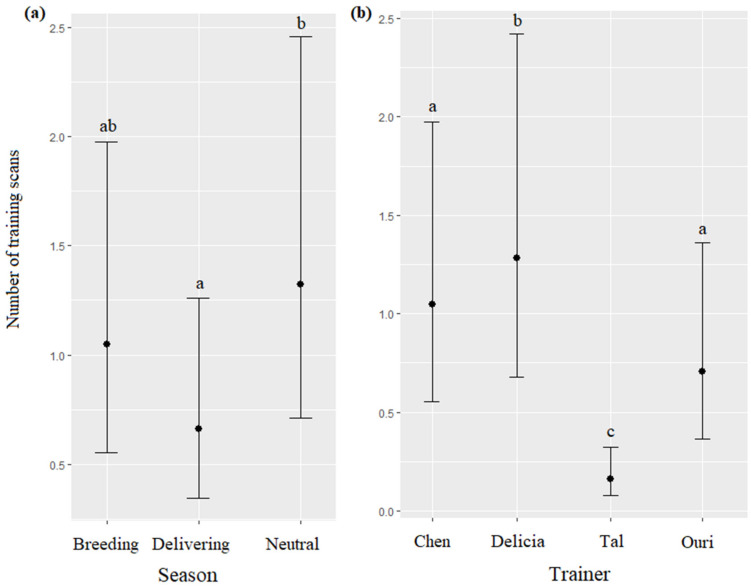
Means and 95% confidence intervals of the number of training scans depending on (**a**) the season and (**b**) the trainer; different letters indicate significant differences (Wald chi-squared test with Bonferroni correction).

The season and trainer significantly impacted the number of training scans (respectively, χ^2^ = 44.71, df = 2, *p* < 0.0001; χ^2^ = 186.64, df = 3, *p* < 0.0001): training scans were more frequent during the neutral season than during the delivering season (Figure 6a), and the most frequent when the trainer was Delicia, followed by Chen, Ouri, and Tal, respectively (Figure 6b, Table 7). The time, age, and sex of the dolphins did not significantly influence the number of training scans (respectively, χ^2^ = 5.77, df = 2, *p* = 0.0557; χ^2^ = 2.41, df = 1, *p* = 0.1204; χ^2^ = 3.10, df = 1, *p* = 0.0780).

#### 3.5.2. Petting Activity

**Figure 7:**
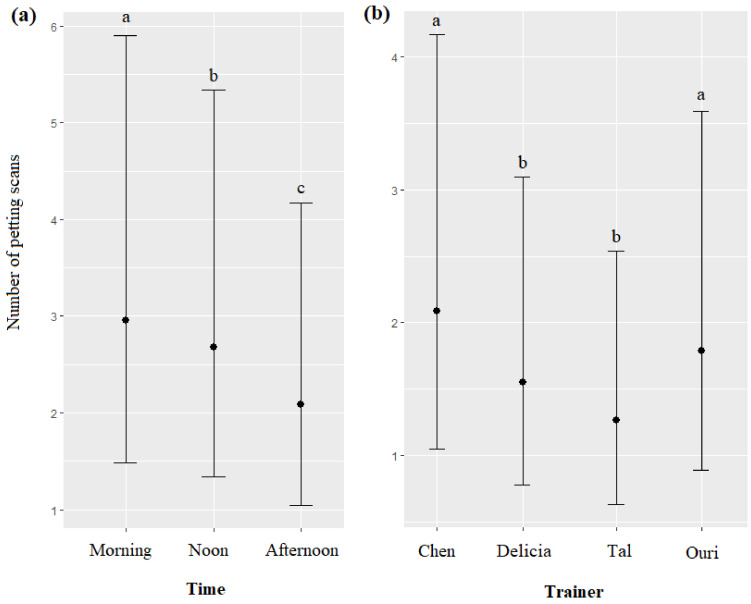
Means and 95% confidence intervals of the number of petting scans depending on (**a**) the time, (**b**) and the trainer; different letters indicate significant differences (Wald chi-squared test with Bonferroni correction).

The time and the trainer’s identity significantly impacted the number of petting scans (respectively, χ^2^ = 48.83, df = 2, *p* < 0.0001; χ^2^ = 74.35, df = 3, *p* < 0.0001): petting scans were more frequent in the morning, followed by noon and afternoon diel intervals (Figure 7a), and more frequent for Chen and Ouri, than for Delicia and Tal (Figure 7b, Table 7). The season, age, and sex of dolphins did not significantly influence the number of petting scans (respectively, χ^2^ = 1.72, df = 2, *p* = 0.4420; χ^2^ = 1.57, df = 1, *p* = 0.2095; χ^2^ = 1.23, df = 1, *p* = 0.2664).

#### 3.5.3. Playing Activity Initiated by the Dolphins with Objects Found in the Facility

**Figure 8:**
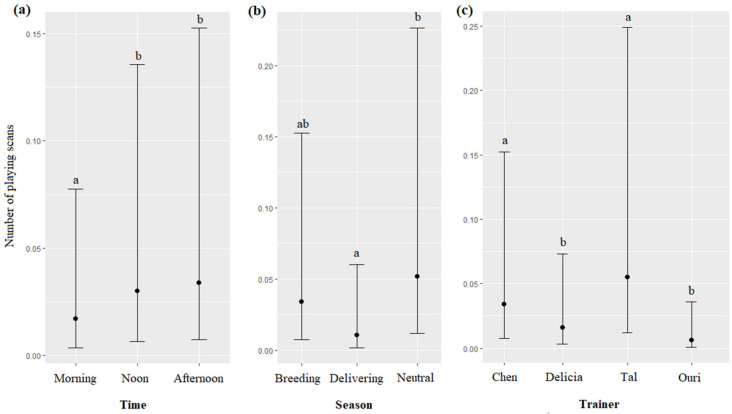
Means and 95% confidence intervals of the number of playing scans depending on (**a**) the time, (**b**) the season, and (**c**) the trainer; different letters indicate significant differences (Wald chi-squared test with Bonferroni correction).

The time, season, and the trainer’s identity significantly impacted the number of the playing scans (respectively, χ^2^ = 12.13, df = 2, *p* = 0.0023; χ^2^ = 12.94, df = 2, *p* = 0.0015; χ^2^ = 45.81, df = 3, *p* < 0.0001): playing scans were more frequent at noon and in the afternoon than during the morning (Figure 8a), more frequent during the neutral season than during the delivering season (Figure 8b), and more frequent for Tal and Chen than for Delicia and Uri (Figure 8c, Table 7). The age and sex of dolphins did not significantly influence the number of playing scans (respectively, χ^2^ = 0.58, df = 1, *p* = 0.4426; χ^2^ = 3.08, df = 1, *p* = 0.0791).

#### 3.5.4. Other Activities

**Figure 9:**
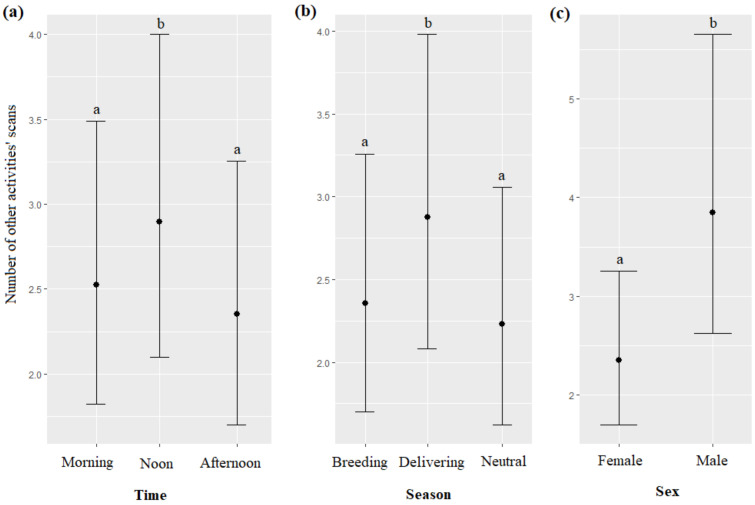
Means and 95% confidence intervals of the number of other activities’ scans depending on (**a**) the time, (**b**) the season, and (**c**) the dolphins’ sex; different letters indicate significant differences (Wald chi-squared test with Bonferroni correction).

The time, season, and sex of the dolphins significantly impacted the number of “other activities” scans (respectively, χ^2^ = 20.88, df = 2, *p* < 0.0001; χ^2^ = 23.61, df = 2, *p* < 0.0001; χ^2^ = 5.44, df = 1, *p* = 0.0196): other activities" scans were more frequent at noon than in the morning, and in the afternoon (Figure 9a), more frequent during the delivering season than during the breeding, and neutral seasons (Figure 9b), and more frequent for males than for females (Figure 9c, Table 7). The trainer’s identity and the age of the dolphins did not significantly influence the number of other activities’ scans (respectively, χ^2^ = 9.93, df = 3, *p* = 0.0566; χ^2^ = 0.01, df = 1, *p* = 0.9031).

#### 3.5.5. Waiting Behavior

**Figure 10:**
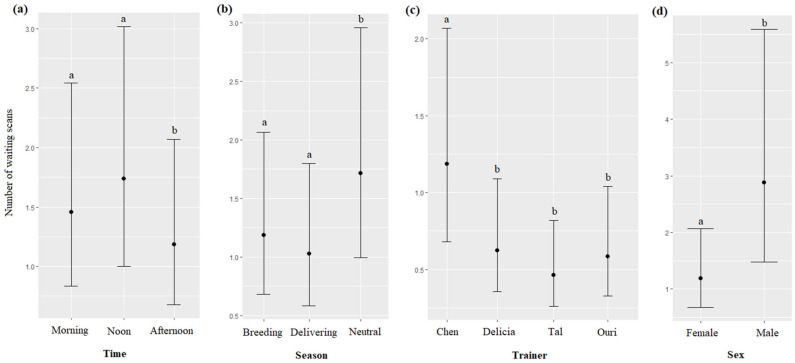
Means and 95% confidence intervals of the number of waiting scans depending on (**a**) the time, (**b**) the season, (**c**) the trainer, and (**d**) the dolphins’ sex; different letters indicate significant differences (Wald chi-squared test with Bonferroni correction).

The time, season, trainer’s identity, and sex of the dolphins significantly impacted the number of the waiting scans (respectively, χ^2^ = 35.12, df = 2, *p* < 0.0001; χ^2^ = 50.53, df = 2, *p* < 0.0001; χ^2^ = 208.10, df = 3, *p* < 0.0001; χ^2^ = 5.65, df = 1, *p* = 0.0174): waiting scans were more frequent at noon than in the morning, and in the afternoon (Figure 10a); more frequent during the neutral season than during the breeding and delivering seasons (Figure 10b); more frequent for Chen than for Delicia, Tal and Uri (Figure 10c), and more frequent for males than for females (Figure 10d, Table 7). The age of the dolphins did not significantly influence the number of waiting scans (χ^2^ = 2.61, df = 1, *p* = 0.1055).

### 3.6. TDIs during Sessions with Toy(s) Provided by the Trainers

During the TDIs sessions involving toy(s) provided by the trainers, dolphins spent most of the time engaged in “other activities” (64.7%), followed by “object play” (35.3%). Among the object play categories, dolphins were engaged 73.7% of the time in “social trainer-dolphin-play”, followed by “individual play” (13,4%), “social play-dolphin” (9.8%), and “stealing toy” (3.1%).

**Figure 11:**
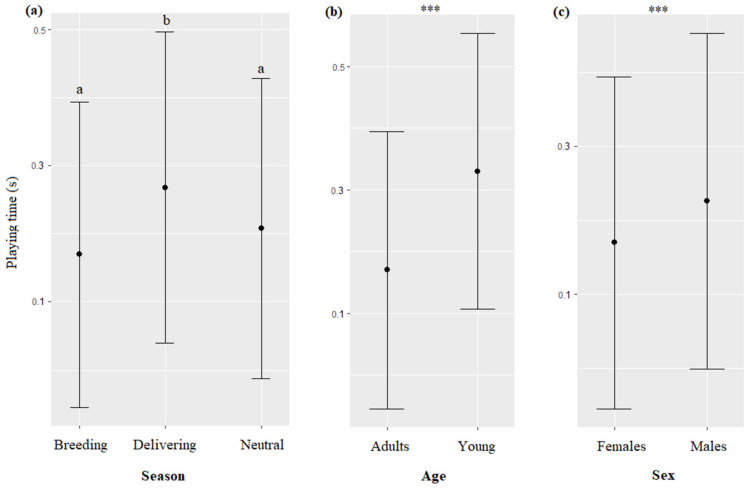
Means and 95% confidence intervals of the time spent playing depending on (**a**) the season, (**b**) the dolphins’ age, and (**c**) the dolphins’ sex. ***: *p* < 0.0001. For variables with more than two levels, different letters indicate significant differences (Wald chi-squared test with Bonferroni correction).

The season, age, and sex of the dolphins significantly impacted the time spent by the animals playing with trainers when toys were provided (respectively, χ^2^ = 9.33, df = 2, *p* = 0.0094; χ^2^ = 168.49, df = 1, *p* < 0.0001; χ^2^ = 76.8, df = 1, *p* < 0.0001): they played more during the delivering season than during the neutral and breeding seasons (Figure 11a), young played more than adults (Figure 11b), and males more than females (Figure 11c, Table 7). The type of interaction, time, trainer’s identity, and initiation and ending modality of the sessions did not significantly influence the time spent by the dolphins playing with trainers (respectively, χ^2^ = 0.19, df = 5, *p* = 0.9992; χ^2^ = 0.58, df = 2, *p* = 0.7476; χ^2^ = 0.59, df = 3, *p* = 0.7532; χ^2^ = 0.13, df = 1, *p* = 0.7167; χ^2^ = 0.03, df = 1, *p* = 0.8454).

**Table 7:** Statistical output from pairwise comparisons for the variables with more than two levels.

Variable	Pairwise Comparison	Participation of the Dolphins	Number of Dolphins Participating to the Sessions	Latency of Response	Number of Training Scans	Number of Petting Scans	Number of Playing Scans	Number of Other Activities’ Scans	Number of Waiting Scans
Time	Morning-Noon	NS	F = 3.54, *p* = 0.0603	NS	NS	χ² = 7.31, *p* = 0.006 *	χ² = 22.24, *p* < 0.0001 *	χ² = 8.65, *p* = 0.0032 *	χ² = 3.01, *p* = 0.0823
	Morning-Afternoon		F = 6.71, *p* = 0.0098 *			χ² = 46.84, *p* < 0.0001 *	χ² = 23.36, *p* < 0.0001 *	χ² = 1.69, *p* = 0.1931	χ² = 9.07, *p* = 0.0025 *
	Noon-Afternoon		F = 0.53, *p* = 0.4679			χ² = 22.24, *p* < 0.0001 *	χ² = 0.41, *p* = 0.5175	χ² = 18.08, *p* < 0.0001 *	χ² = 39.74, *p* < 0.0001 *
Season	Breeding-Delivering	χ² = 0.76, *p* = 0.3808 *	F = 0.98, *p* = 0.3204	NS	χ² = 2.22, *p* = 0.1359	NS	χ² = 4.94, *p* = 0.0262	χ² = 17.21, *p* < 0.0001 *	χ² = 0.50, *p* = 0.4771
	Breeding-Neutral	χ² = 26.14, *p* <0.0001 *	F = 19.99, *p* < 0.0001 *		χ² = 5.53, *p* = 0.0186		χ² = 3.65, *p* = 0.0558	χ² = 2.83, *p* = 0.0920	χ² = 22.45, *p* < 0.0001 *
	Delivering-Neutral	χ² = 47.39, *p* < 0.0001 *	F = 48.32, *p* < 0.0001 *		χ² = 42.20, *p* < 0.0001 *		χ² = 10.36, *p* = 0.0012 *	χ² = 23.03, *p* < 0.0001 *	χ² = 40.36, *p* < 0.0001 *
Trainer	Delicia-Chen	χ² = 3.53, *p* = 0.0600	F = 14.05, *p* = 0.0002 *	χ² = 16.97, *p* < 0.0001 *	χ² = 8.83, *p* = 0.0029 *	χ² = 36.15, *p* < 0.0001 *		χ² = 5.48, *p* = 0.0191	χ² = 112.01, *p* < 0.0001 *
	Delicia-Tal	χ² = 6.05, *p* = 0.0014 *	F = 45.66, *p* < 0.0001 *	χ² = 20.11, *p* < 0.0001 *	χ² = 126.57, *p* < 0.0001 *	χ² = 1.23, *p* = 0.2667	χ² = 26.53, *p* < 0.0001 *	χ² = 0.01, *p* = 0.8996	χ² = 5.23, *p* = 0.0220
	Delicia-Uri	χ² = 38.80, *p* < 0.0001 *	F = 34.49, *p* < 0.0001 *	χ² = 1.18, *p* = 0.2760	χ² = 34.55, *p* < 0.0001 *	χ² = 7.35, *p* = 0.0066 *	χ² = 3.66, *p* = 0.0554	χ² = 0.62, *p* = 0.4293	χ² = 0.17, *p* = 0.6733
	Chen-Tal	χ² = 7.79, *p* = 0.0052 *	F = 14.98, *p* = 0.0001 *	χ² = 4.42, *p* = 0.0354	χ² = 131.54, *p* < 0.0001 *	χ² = 59.97, *p* < 0.0001 *	χ² = 6.16, *p* = 0.0130	χ² = 3.65, *p* = 0.0558	χ² = 134.95, *p* < 0.0001 *
	Chen-Uri	χ² = 9.69, *p* = 0.0018 *	F = 12.7651, *p* = 0.0004 *	χ² = 0.14, *p* = 0.7045	χ² = 5.90, *p* = 0.0150	χ² = 4.07, *p* = 0.0434	χ² = 14.66, *p* = 0.0001 *	χ² = 2.01, *p* = 0.1560	χ² = 65.86, *p* < 0.0001 *
	Tal-Uri	χ² = 2.40, *p* = 0.1056	F = 0.59, *p* = 0.5668	χ² = 0.24, *p* = 0.6658	χ² = 69.85, *p* < 0.0001 *	χ² = 33.58, *p* < 0.0001 *	χ² = 21.56, *p* < 0.0001 *	χ² = 2.15, *p* = 0.1425	χ² = 5.18, *p* = 0.0227

* significant differences for variables with more than two levels.

## 4. Discussion

Only a few studies have investigated non-domesticated animals’ motivation to interact with humans and how much they might value this exchange [12,14,41,67,68]. The assessment of the interactions between the trainers and the dolphins in a controlled environment has recently been shown to be a useful tool to evaluate the welfare of the animals [14,41,68]. In addition, the particular trainer-dolphin relationship developed through repeated positive interactions may also explain the animals’ motivation to interact with their caretakers [7,41,69]. However, because the interactions between the dolphins and their trainers are usually reinforced by food, it is difficult to distinguish the drive of the animals to interact with their caretakers from the food drive. Therefore, the unique management adopted by TDR, where dolphins can choose whether or not to interact with their trainers in the absence of food rewards, allowed a more objective assessment of the interactions between the animals and their caretakers. Since the willingness to interact with the trainers has been shown to be linked with the dolphins’ welfare, the results obtained in this study could be used to draw possible conclusions regarding the well-being of TDRs bottlenose dolphins [14].

### 4.1. Dolphins’ Overall Participation

Overall, TDRs dolphins exhibited a high participation rate during TDIs sessions, with an average of three dolphins participating per session. The “*call*” that was performed by the trainers to inform the dolphins of their availability to interact with them was not linked with a higher participation rate of the animals in the sessions. The dolphins responded more frequently, and their latency of response was shorter on average during the “*no-call*” (latency 0; 0–1 min) than with the “*call*”, even though the latter still showed a short latency of response (less than 1 min in average). Considering both the dimension of the facility (14,000 sqm) and the tourist activities that took place at TDR during the day that could distract the dolphins from interacting with their trainers, the quicker response of the animals, either after the “call” or “no-call”, might indicate a high motivation to participate to the TDIs. The results regarding the use of the “*call*” and “*no-call*” by the trainers might have probably been strongly impacted by the predictable time schedules followed by the caretakers at TDR, which dolphins may have been familiar with. In fact, it was not unusual to see the dolphins following the trainers from the edge of the enclosure to the platforms, therefore not enabling the caretakers to perform the “*call*”. This type of behavior, which can be referred to as “anticipatory behavior", has already been observed in other studies on captive bottlenose dolphins and indicated the motivation of animals to participate in the upcoming activity with its trainers [14,41]. Since no food was provided during the TDIs at TDR, the dolphins were only anticipating the interaction with their trainers, therefore underlining the willingness of the animals to participate in such activities. The regularity and recurrence of the TDIs for daily management and care probably allowed the animals to get well acquainted with their trainers, leading to the development of a HAR (human-animal-relationship) [8,9,10,70], which could explain the bottlenose dolphins’ motivation to interact with the caretakers without food rewards. This result seems to confirm that positive HAI (human-animal interaction) can represent gratifying events for animals who are therefore motivated to participate in the activities proposed by familiar humans [7,27,71].

On the other hand, the four trainers selected for the study were also the same who fed the dolphins during the feeding sessions. Therefore, a link between the trainers and the food provisioning was still present at TDR, potentially influencing the time the dolphins spent interacting with their caretakers. Nevertheless, it is important to underline that the strict separation between the feeding times and the other interactions with the trainers was already in place for years at TDR (at least more than ten years before the start of the current study). Therefore, dolphins were most likely used to this routine, whether they were born within the facility or not, and they understood no food would have been provided outside of the feeding sessions. Thus, the hypothesis of an operant conditioning mechanism that would have associated the presence of the trainers with the food rewards does not represent an explanation for the high participation of the bottlenose dolphins in the TDIs at TDR. On the other hand, it could be suggested that the dolphins’ participation in the TDIs might be mostly explained by the type of activities provided by the trainers, such as play, petting, and training, which could have been attractive for the animals, or satisfied the individuals’ needs for social interaction outside the dolphin group.

Moreover, the TDIs sessions where the trainers used toy(s) received higher rates of attendance, high frequency of response (independently of the modality of the “*call*” or “*no-call*”), and with higher numbers of dolphins participating compared to the TDIs without toys. Both captive and wild dolphins engage in a variety of play activities with other individuals, including object play [55]. In some cases, the presence of the humans seems to stimulate an increase in play behaviors among dolphins [39], while the presence of the toys has been shown to elicit more social play among the animals [72,73]. The results obtained here confirm that the combination of interaction with the trainers and the toys might be more attractive to the dolphins than when these two elements are proposed separately to them [58]. This seems to be confirmed by the fact that the play activities during the TDIs with toy(s) were characterized by a higher frequency of social play between the trainers and the dolphins compared to the other types of play categories. The social play between the trainers and the dolphins at TDR was mostly characterized by tug-of-war, chasing after the trainer who held the toy, and play-fetch with balls or long bandanna, with the dolphins bringing the objects back, and relinquishing it to their caretakers. Even though bottlenose dolphins have been observed engaging in intra-specific object play activities before [74], only another study confirmed the presence of actual social object play among dolphins, where one individual relinquished the object in favor of the other one [75]. Once again, since no food was provided during these sessions, these results might reflect the higher motivation of the dolphins to simultaneously play with the toys and the trainers. This information may be valuable for the captive facilities whose aim is to improve the welfare of their dolphins, and the relationship between the trainers and the animals.

### 4.2. Dolphins’ Preference for the Different Trainers

In general, the bottlenose dolphins at TDR showed a high participation rate, short latency of response, and with more individuals engaged in the TDIs when the trainers were Delicia and Hen. Delicia was more of a “dynamic" type of person during the sessions (running from one platform to another one, followed by the dolphins; jumping in the water), which might have been appreciated by certain dolphin individuals, explaining their high attendance during her activities. On the other hand, Hen had a more “nurturing” attitude, and was more prone to pet the dolphins upon “their request” and to follow “their mood” on the activity they wanted to perform with her. It must be noted that Delicia and Hen were the trainers who performed most of the TDIs, and they were the only two caretakers available to provide toy(s) during the sessions, which might have biased the current results, and the HAR that had been established between them and the dolphins. 

Moreover, the dolphins seemed also to exhibit preferences toward certain trainers during specific activities. For example, the bottlenose dolphins at TDR initiated more play activities (characterized by the dolphin bringing an object they found in the facility, such as a piece of plastic or a dead jellyfish, to the trainers) with Tal. Tal was the trainer who spent most of the time talking to the tourists, but she often used to stop this activity to give attention to the dolphins when they brought to her things they found to initiate play. Petting and waiting activities were also performed more often by the dolphins during Hen’s sessions. As mentioned above, Hen had a more “nurturing” personality, potentially explaining the higher frequency of petting and the willingness of the dolphins to wait for her attention. 

The dolphins might have developed a preference towards one trainer or another depending on both the specific attitude of the caretakers during the sessions and/or the activities he/she was proposing. In general, it is well known that both humans and dolphins have different personalities, experiences, and life histories, all of which can modulate their social daily life [76,77,78,79]. The animals’ life experiences might influence the development of the preferences for specific trainers and vice-versa, which might modulate the HARs between the parties involved [14]. In the current study, each dolphin’s preference was not analyzed, and no personality tests were performed on dolphins or trainers. Therefore, further research should be conducted to better understand HAR between the dolphins and their trainers, and to find out if the animals develop real preferences towards specific caretakers and what parameters influence them.

### 4.3. Diel and Seasonal Variations in the Dolphins’ Participation

No clear diel pattern was found in the dolphins’ participation during the TDIs, with similar attendance rates for the three time intervals considered, even though more dolphins seemed to participate in the morning sessions. Studies on cetacean species showed that captive animals, including dolphins, are most active in the morning [73,80], which could explain the result obtained in the current study. Moreover, high scores of dolphins’ participation in a specific activity seemed to be linked to particular diel intervals. Precisely, the dolphins spent more time engaged in petting in the morning, playing in the afternoon, and being involved in “other” activities and waiting for the trainers’ attention at noon. These patterns may have been linked with the dolphins’ daily rhythms and the schedule of the activities occurring at the facility. Dolphins may have adapted the interactions they engaged with their trainers depending on their surrounding environment. For instance, most tourist activities in the water occurred at noon and in the afternoon at TDR. It is well known from previous studies that variations in the social behaviors of dolphins can occur in relation to the presence of visitors in the facility [81,82]. Therefore, it is possible that TDRs bottlenose dolphins might have searched for the attention of the trainers (familiar humans) to cope with potentially unwanted interactions with unknown people, resulting in more play and waiting behaviors during the noon and afternoon diel intervals. On the other hand, petting, for instance, might have been appreciated by the dolphins in a quiet environment that corresponded to the morning time at TDR. Petting, besides being used as a “secondary reinforcement” at TDR, was also often initiated by the dolphins who approached the trainers and presented them with the part of the body the animals wanted to be petted (i.e., the dolphins approaching the trainers directly with the belly up). In general, the bottlenose dolphins have very sensitive skin, with the sensitivity to be close to that of humans in the most sensitive skin areas, such as the tactile surface of the fingers, the skin of the eyelids, and the lips [50]. The high skin sensitivity in dolphins might also be the cause of the animals appreciating the contact with their trainers. In horses, a manual imitation of grooming of certain parts of the body by humans has been proven to produce a reduction of the heart rate as a consequence of the stimulation of the release of β-endorphins [83,84,85]. The same physiological process could occur in the dolphins, who, like horses, use body contacts to establish and maintain social bonds. Therefore, the bottlenose dolphins at TDR might have used petting as a way to reinforce their relationship with their trainers, for example, to reconnect with them after the long nighttime interval of separation from 4 pm to 9 am, or simply for personal satisfaction (i.e., pleasure), or both. These results suggest that positive HAR that involves voluntary body contacts might be rewarding for dolphins like they are for other animal species [51].

The seasonal variations during the trainer-dolphins interactions showed a clearer pattern, with the participation rate, response to the trainers’ presence (independently of the modality of the “*call*” and “*no-call*”), and the number of dolphins participating in the TDIs being higher during the neutral season, and lower during the other two seasons considered. Dolphins also spent more time engaged in training and waiting activities, and initiating play with the trainers during the neutral season compared to the other periods of the year. The breeding and delivering seasons are characterized by obvious hormonal [40] and social changes in the group of the dolphins. These changes might have made the dolphins more motivated to engage in intraspecific social behaviors, including socio-sexual or maternal behaviors, which could have distracted them from interacting with their trainers [42]. In contrast, the neutral season was the time of the year when no breeding activities or births happened, leaving the dolphins with more time to engage in interspecific interaction with their trainers. On the other hand, dolphins spent more time in “other” activities and object play during the delivery season. During the current study, the delivery season was characterized by the birth of four calves (Pashosh’s calf died a few days later the birth), which might have kept four of the five adult females occupied. The four juveniles, whose mothers were nursing newborns, might have had more time and motivation to interact with the trainers during the delivery season, including playful and “other” activities. These seasonal changes in the dolphins’ participation during the trainers’ sessions should be also considered when using such indicators to assess dolphins’ welfare.

### 4.4. Sex and Age Classes Differences in Dolphins’ Participation

Sex and age class differences were observed during the TDIs. Males responded quicker and more often during the TDISs than females. Males also spent more time “waiting” for the trainers’ attention and played more with trainers when toy(s) were provided, the latter result was also reported by a previous study [74]. These results could be explained by the fact that the two animals exhibiting the highest amount of time spent with the trainers were two males, one adult (Shandy), and one young (Suf). In addition, two adult males (Shandy and Lemon) were separated from the rest of the group every day from 9 am to 4 pm. These two individuals might have increased their interest to interact with the trainers as a substitution for the lack of interaction with their conspecifics. 

Furthermore, young dolphins played more than adults during sessions involving toy(s), which is in line with the fact that young individuals tend to play more than adults [49]. Young dolphins may have been more motivated than adults to participate when toy(s) were provided. Since our sample size was limited (9 females and 5 males; 8 adults and 6 young), the reported age and sex differences may strongly be linked to personality, and our hypotheses cannot be validated.

### 4.5. Inter-Individual Differences in Participation and Health/Welfare Implications

All the results that have been presented and discussed here have been collected on a small number of dolphins, and some of them might have been strongly linked with inter-individual differences. Each dolphin exhibited a particular participation rate and engaged differently in the activities proposed by the trainers, which might have been linked with individual features such as personality, social rank, or reproductive status. Another parameter that may have played a role in the participation during the TDIs is the health/welfare status of the dolphins. In the current study, the cases of Mika and Shy are good examples to demonstrate the potential links between participation in the interactions with the trainers, dolphin personality, and their health/welfare. Mika (young female) and Shy (adult female) were the dolphins with the lowest participation rate, and lowest time spent with the trainers. Mika was a three-year-old juvenile female that died seven months after the start of the study (the cause of the death remains unclear). According to the trainers’ records of the dolphins’ food intake, the appetite of Mika decreased during the seven months before her death. The “*Willingness to participate*” (WtP) in the training sessions has already been shown to be an indicator of captive dolphins’ welfare and to be a more useful tool for health monitoring than food intake because its frequency decreases even before the animal’s appetite drops [86]. In fact, other anecdotal evidence provided by a previous study confirmed that dolphins’ participation in training sessions decreases before the deterioration of their health conditions is diagnosed [87]. A decreased motivation to work for food rewards could therefore be correlated with a deterioration of the health and/or welfare of captive dolphins [87,88,89,90]. Since feeding and interactions were separated at TDR, it could be easily said that both Mika’s appetite and her motivation to interact with the trainers decreased, indicating a potential deterioration of the HAR. However, the participation of Mika was only assessed seven months before her death; therefore, her participation rate during healthy conditions was not known, making it hard to validate our hypothesis. The following two other dolphins died during the study: an adult female, Domino, whose death was sudden and without any previous behavioral and physiological changes, and her male calf, Bar, who died a couple of weeks after his mother. The cause of Domino’s death was disclosed by TDR, but her health and welfare were fine before her death, explaining her good participation during the sessions. It was not possible to assess the participation of Bar during the sessions after his mother’s death because he was separated from the rest of the group upon this event.

On the other hand, Shy, a healthy adult female, showed among the lowest participation rates during the TDIs. In this case, the low participation was probably not related to the health status of the animal since Shy was healthy but might have been linked to her personality. According to the trainers’ assessment, Shy was, as per her name, a timid dolphin who was not participating as often as the other individuals in the sessions that took place at the platforms, but she was quite keen to accompany the trainers whenever they were swimming around the facility (trainers’ personal communication). The timid personality of this dolphin might also have influenced her calf (Nikita), who was “not allowed” by her mother to spend time with the trainers at the platforms till she was weaned (indicated also by low participation scores) (trainers’ personal communication). Considering all the presented cases, we suggest that dolphins might have exhibited their own level of motivation to interact with the trainers, which could be linked to their personality. In addition to that, for each individual dolphin, the motivation to interact with the trainers might be modulated by the individual’s health and welfare status. Therefore, it is important to daily monitor the participation of dolphins during sessions when they are healthy in order to obtain baseline data for each individual and detect any unexpected drop in this participation that could indicate a major health or welfare issue.

## 5. Conclusions

The present study aimed to answer the following three questions: are dolphins under human care motivated to interact with their trainers in the absence of food rewards? If so, why do dolphins want to interact with their known trainers, and what parameters modulate their motivation? The dolphins at TDR showed a high level of participation in the sessions proposed by the trainers, which clearly shows their motivation to interact with their caretakers without any food reward. This motivation was even stronger when toys were provided by trainers during these TDIs. The dolphins’ motivation to interact with the trainers was modulated by the time of the day, the season, the trainer’s identity, the dolphin’s age, sex, and probably the dolphin’s personality. The current results also showed that the “*call*” performed by the trainers was not critical in determining the response of the animals. In fact, the dolphins might have anticipated the trainers’ sessions since they occurred on a fixed schedule, and the animals could have been waiting for their caretakers, making them unable to perform the “call”. 

Regarding the reason why dolphins choose to interact with the trainers in the absence of food rewards, several hypotheses can be made. Boredom may be one of the reasons explaining the high participation. Even though many activities occurred at TDR, including feedings, activities with the public, and social interactions among the dolphins, the animals might still have experienced boredom, and the interactions with trainers might have been a way to distract themselves. Another hypothesis explaining the participation of dolphins is the probable formation of a relationship between the trainers and the dolphins that could be close to the concept of “friendship” A “*friendship*” could arise from a history of mostly positive interactions between two individuals, which can be measured with affiliative behaviors, such as body contacts (i.e., grooming, petting), seeking the proximity of each other, and spending time in close contact [10,13,91,92]. In the current study, the dolphins often engaged in body contact with their trainers and exhibited a high frequency of associations/interactions with them, therefore fulfilling the “friendship” criteria. In addition, Hediger [93] stated that a series of positive HAIs might lead the animals involved to perceive the humans they interact with as conspecifics through a long-term process referred to as “*assimilation tendency*”, which enables the humans to get “accepted” as a member of the animal group. This kind of HAR could explain the motivation of dolphins to interact with their trainers to maintain and strengthen the bond between them. In a place such as TDR, where dolphins and trainers share the same social environment, the management might satisfy both the fission and the fusion aspects that are characteristics of the social lives of bottlenose dolphins [39].

Finally, the case of Mika, whose participation was very low before her death, might confirm that the willingness to interact with the trainers could be used as a measure to assess the health/welfare of the dolphins under human care [14,41] and in this specific case, without any link with food provision. Therefore, it could be suggested that the HAIs should be closely monitored in the facilities that keep captive dolphins. Individual-based studies should also be conducted to better understand the relationships between specific trainers and specific dolphins and to evaluate the strength of their relationships. A better understanding of the trainer-dolphin relationship may enable facilities to improve the dolphins’ social environment and, thus, their welfare.

## Figures and Tables

**Table 5:** Variables used for the statistical analysis (A: adult; Y: young; F: female; M: male).

	Sampling Method	Variables	Content
Response variables	Continuous recording and scan sampling	Participation of dolphins	Participation/No participation
	Continuous recording and scan sampling	Participation in the session	Number of dolphins participating
	Continuous recording and scan sampling	Latency of response	0/0–1 min/2–4 min/5–10 min/more than 10 min
	Scan sampling	Training	Number of scans spent in training activity
	Scan sampling	Contact	Number of scans spent in contact with the trainer
	Scan sampling	Petting	Number of scans spent being pet by the trainer
	Scan sampling	Playing	Number of scans playing with the trainer
	Scan sampling	Waiting	Number of scans waiting for the trainer
	Continuous recording (sessions with toys only)	Time spent playing	Time spent playing with toy(s)
Predictors	Continuous recording and scan sampling	Time	Morning/Noon/Afternoon
	Continuous recording and scan sampling	Season	Breeding/Delivering/Neutral
	Continuous recording and scan sampling	Trainer	Delicia/Chen/Tal/Ouri
	Continuous recording and scan sampling	Type of session	Without toy(s)/With toy(s)
	Continuous recording and scan sampling	Dolphin Sex	Female/Male
	Continuous recording and scan sampling	Dolphin Age	Adult/Young
	Continuous recording and scan sampling	Call modality	Call/No call
	Continuous recording and scan sampling	Call duration	Duration
	Continuous recording (sessions with toys only)	Type of interaction	Individual play/Stealing toy/Social play-dolphin/Social play-trainer/Other activity
	Continuous recording (sessions with toys only)	Object play Initiation modality	Dolphin/Trainer
	Continuous recording (sessions with toys only)	Object play Ending modality	Dolphin/Trainer

**Table 6:** Participation rates, and percentage of session duration spent with the trainer for each studied bottlenose dolphin (the percentages in *italic* are the two lowest, and percentages in **bold** are the two highest for each variable).

Individual	Age	Sex	Participation Rate (Percentage of the Total Number of Sessions)	Time Spent with the Trainer (Percentage of Total Sessions’ Duration)
Cindy	Adult	M	25.4%	7.3%
Dana	Adult	F	25.1%	11.6%
Domino	Adult	F	15.0%	6.6%
Pashosh	Adult	F	30.6%	11.1%
Shy	Adult	F	*11.7%*	*3.0%*
Lemon	Subadult	M	**63.8%**	26.1%
Shandy	Subadult	M	**62.0%**	**41.7%**
Nana	Subadult	F	27.3%	21.8%
Suf	Juvenile	M	42.5%	**30.2%**
Luna	Juvenile	F	32.9%	17.9%
Mika	Juvenile	F	*11.0%*	*4.5%*
Jampa	Juvenile	F	35.0%	18.7%
Bar	Calf	M	23.2%	7.2%
Nikita	Calf	F	23.3%	10.2%

## Data Availability

Data can be required upon direct contact with the corresponding author of this paper.
